# A Population-Based, Cross-Sectional Study Examining Health Services Deficits of US Veterans Using 2014 Behavioral Risk Factor Surveillance System Data: Is Rural Residency an Independent Risk Factor after Controlling for Multiple Covariates?

**DOI:** 10.3390/healthcare5030039

**Published:** 2017-07-31

**Authors:** Catherine A. St. Hill, Michael T. Swanoski, Martin S. Lipsky, May Nawal Lutfiyya

**Affiliations:** 1 Department of Experimental and Clinical Pharmacology, College of Pharmacy, University of Minnesota, Minneapolis, MN 55455, USA; sthil001@umn.edu; 2 Department of Pharmacy Practice and Pharmaceutical Sciences, College of Pharmacy, University of Minnesota, Minneapolis, MN 55812, USA; mswanosk@d.umn.edu; 3 Roseman University of Health Sciences, South Jordan, UT 84095, USA; mlipsky@roseman.edu; 4 National Center for Interprofessional Education and Practice, Children’s Rehabilitation Center, University of Minnesota, Minneapolis, MN 55455, USA

**Keywords:** rural US veterans, Health Services Deficits, BRFSS data, chronic illness

## Abstract

**Introduction:** In 2014, it was reported that there was a backlog of an estimated 1.2 million claims nationwide at the United States Veterans Administration (VA). This ecological occurrence opened up a space for asking and answering some important questions about health service deficits (HSD) of US veterans, which is the focus of the research reported on in this paper. The purpose of this study was to ascertain if rural veterans were more likely to experience HSDs than urban military veterans after controlling for a number of covariates. **Methods:** Bivariate and multivariate data analysis strategies were used to examine 2014 Behavioral Risk Factor Surveillance System (BRFSS) survey data. HSD was the dependent variable. **Results:** Two multivariate models were tested. The first logistic regression analysis yielded that rural veterans had higher odds of having at least one HSD. The second yielded that rural US veterans in 2014 who had higher odds of having at least one HSD were: 18–64 years of age, unemployed seeking employment, living in households with annual incomes lower than $75,000, without a university degree, not part of a married or unmarried couple, a current smoker, and/or a binge drinker within the last 30 days. **Conclusions:** The study described here fills identified epidemiological gaps in our knowledge regarding rural US military veterans and HSDs. The findings are not only interesting but important, and should be used to inform interventions to reduce HSDs for rural veterans.

## 1. Introduction

Since 1990, the United States (US) has engaged in multiple major conflicts at distinct periods of time. Starting in 1990, the US was involved in the first Gulf War, termed Operation Desert Storm [1]. A decade later, in 2001, the US began its most recent involvement in the Middle East with the invasion of Afghanistan in Operation Enduring Freedom (OEF) [1] and later in 2003 in Iraq in Operation Iraqi Freedom (OIF) [1]. The first phase of Operation Enduring Freedom ended in 2014; however, the US remains involved in Afghanistan as part of a North American Treaty Organization (NATO) led initiative. Presently, more than 1.6 million American men and women have served or are currently serving in support of the NATO Afghanistan initiative, OEF, and OIF [2,3].

In 2014, it was reported that there was a backlog of an estimated 1.2 million claims nationwide at the United States Veterans Administration (VA) [4]. Moreover, wait times to be seen by a healthcare provider extended well past the legislated 30 days [5]. Few would deny that the VA has long struggled to cope with claims of all sorts [6]. The backlog reported, however, had been amplified as more soldiers needing a wide array of healthcare returned from Iraq and Afghanistan [7]. Since 2014 there has been considerable press coverage regarding the manner in which the VA has either managed or mismanaged their claims backlog. This ecological occurrence has opened up a space for asking and answering some important questions about health service deficits (HSD) of US veterans, which is the focus of the research reported on in this paper.

HSD [8,9,10] is an evolving concept and is composed of the following: no routine medical exam, no primary care provider, no health insurance, and/or deferring medical care because of cost; if one at least one of these has been experienced within the last 12 months, this constitutes having an HSD [8,9,10]. These four factors represent HSDs in part because of their direct connection to preventive care and/or access to care. In the US, health insurance is very often a determinant of healthcare access, just as having the financial means for the co-pay portion of care or out-of-pocket charges for healthcare. Annual or routine check-ups and/or having a personal healthcare provider are essential for the receipt of preventive care in the US.

An earlier study [4], analyzing Behavioral Risk Factor Surveillance System (BRFSS) data, examined depression and HSDs in rural compared to non-rural adult populations and found that rural residency was an independent risk factor for greater HSDs in adults with depression. [8] In 2014, a study was published examining the prevalence of HSDs in depressed (determined by a standardized and validated measure) US veterans [11]. Rural residency was found to be an independent risk factor for greater odds of having a HSD in rural veterans. HSDs have also been examined in adults with asthma [10] as well as arthritis [9], with rural geographic locale found to be significantly associated with HSDs in those populations.

In the US, a higher per capita proportion of military veterans come from rural rather than non-rural communities [12]. US military veterans, if discharged or released under conditions other than dishonorable, are eligible to receive healthcare benefits through the Veterans Health Administration (VA). The VA is the US’s largest integrated healthcare system and has defined criteria for the priority of healthcare services for eligible veterans. These priority categories favor military service-related injuries and illnesses [1,2,3,4,5].

Access to care has often been noted as an issue in rural communities for both veterans and non-veterans [13]. The VA has made efforts to expand services to rural areas to ensure adequate access [1,14]. Moreover, in the past decade, greater attention has been paid to veterans’ mental health issues as a direct result of their wartime deployment [2], and subsequent changes in mental health status [2]. For instance, there has been a documented increase in depression, [15,16] post-traumatic stress disorder (PTSD) [17], alcohol and substance abuse [15], and suicide [18] among US military veterans. In response to these emergent issues, the VA has increased mental health staffing [19] and has implemented evidence-based models of integrated mental health services in outpatient clinics [14]. As important as mental healthcare is for veterans, especially for those returning from combat, healthcare for other health-related issues are equally important since these health conditions impact their quality of life. We believe that an assessment of HSDs is necessary for all veterans.

The purpose of this study was to ascertain if rural veterans were more likely to experience HSDs than urban military veterans after controlling for a number of covariates. The study described here sought to fill identified epidemiological gaps in our knowledge regarding rural US military veterans and HSDs. Among the epidemiological gaps are whether or not rural veterans have greater odds of experiencing HSDs in comparison to urban veterans. Furthermore, we do not know if the VA has successfully bridged healthcare access disparities for rural veterans, reducing the possibility of HSDs as we have defined them. Analyzing population-based, non-VA, non-medical records or data for veterans might better identify the population of US military veterans (by specific characteristics) who may have HSDs, thus providing direction for health systems interventions aimed at solutions for reducing HSDs.

## 2. Methods

To answer the research question, 2014 BRFSS data were analyzed using bivariate and multivariate techniques. BRFSS is a random digit telephone survey that is a collaborative project of the Centers for Disease Control and Prevention (CDC) and all US states and territories. The survey measures several behavioral risk factors and disease states in the non-institutionalized US adult population aged 18 through 99 years. A complex multi-stage sampling approach is used by BRFSS and subsequently a weighting factor is calculated for application to the data in order to ensure that they are representative of the US population based on the most recent census data. A more in-depth description of the data weighting process can be found elsewhere [20].

BRFSS collects information from individuals on health risk behaviors, preventive health practices, chronic conditions, and healthcare access primarily related to chronic disease and injury. BRFSS is composed of core questions that must be asked of every survey participant, as well as optional modules that may be chosen by individual states and asked only of the survey respondents from the participating state(s). To identify veterans for this study, responses to the question: *Are You a Veteran*? were used. All respondents selecting *yes* were considered military veterans.

For analysis, a number of variables were either re-coded or computed. Re-coding for the most part entailed collapsing response categories and removing the response categories of *don’t know* and *refused*. Re-coded variables were: age, employment status, marital status, deferment of medical care within the past year because of cost, timing of last routine medical check-up, health insurance status, personal healthcare provider, education attained, annual household income, binge drinking, smoking status, body mass index (BMI), arthritis, depression in lifetime, diabetes, and geographic locale. All variable re-codes were undertaken for clarity of factors for analysis and ease of interpretation. For instance, in BRFSS, marital status has nine categories. These were re-coded into two categories: *Married or Part of Unmarried Couple*, and *Not Married or Part of Unmarried Couple*, because these two categories encapsulate the concepts of interest. Likewise, education had nine original categories that were reduced to three through re-coding. The re-coded categories represent the most meaningful ones for this analysis. Age was re-coded into two categories: *18–64 years of age* and *65 years and older*. This re-code was purposive, since in the US persons 65 years of age and older are covered by Medicare regardless of their military status.

The geographic locale variable was determined using the metropolitan statistical area (MSA) variable provided in the BRFSS database. MSA is comprised of geographic entities delineated by the Office of Management and Budget (OMB) for use by Federal statistical agencies in collecting, tabulating, and publishing federal statistics. The MSA variable was recoded into the dichotomous categories of rural and urban. Rural veterans were defined as people living either within an MSA that had no center city or outside an MSA. Urban veterans included all respondents living in a center city of an MSA, outside the center city of an MSA but inside the county containing the center city, or inside a suburban county of an MSA.

Three variables were computed from other variables in the BRFSS database. These were race/ethnicity, chronic disease, and HSDs. Race/ethnicity was calculated from participant responses to two separate survey questions—one regarding race and the other regarding Latino/Hispanic ethnicity. All race/ethnicity categories were computed as mutually exclusive entities. For example, all respondents coded as Caucasian chose white as their racial classification, likewise, black for African American, etc. If a respondent identified themselves as Hispanic or Latino they were classified by that ethnic category regardless of any additional racial classification. The category of Other/Multiracial was also calculated.

The chronic disease variable was a composite variable calculated from factors from four separate variables—have arthritis, have diabetes, have lifetime depression, and obesity, as measured by the calculated BMI. These were chosen because they are common and age prevalent. Having at least one of these conditions was coded as at least one chronic condition, although this did not preclude respondents from having more than one of these chronic conditions.

As mentioned in the introduction, the variable HSD was computed from the factors of four different variables: no routine medical exam, no primary care provider, no health insurance, and/or deferring medical care because of cost, all within the last 12 months. Having at least one of these was coded as at least one HSD; this did not preclude respondents from having more than one of these. The variable HSD was the dependent variable for the study.

For bivariate analysis, since all of the independent variables were categorical, either a chi square or an odds ratio was computed as the test statistic. For the multivariate analyses performed, an adjusted odds ratio was the calculated test statistic. All analyses were performed on weighted data as is recommended by the CDC. The weighting, calculated by the CDC, uses the most recently available census data to provide a stratified representation of the nation’s non-institutionalized population. Only findings from weighted analyses were considered valid. All analyses were performed using SPSS version 24 (IBM, Chicago, IL, USA) with alpha set at *p* < 0.05. The Institutional Review Boards (IRBs) of the researchers’ institutions recognize that the analysis of de-identified, publicly available data does not constitute human subjects research as defined in federal regulations, and as such does not require IRB review. Human subjects review was not sought nor received.

## 3. Results

Table 1 displays the study variables data describing US veterans as of 2014. Descriptive analysis yielded that in 2014, 20% of US veterans lived in rural geographic locales, 91% were males, and 81% were between the ages of 18 and 64 years. An estimated 26% were university graduates with at least a four-year degree, 47% were employed for wages, and 65% were either married or part of an unmarried couple. While 81% self-reported their health as good to excellent, 61% had at least one chronic disease. Binge drinking in the past 30 days was reported by 15% of veterans, while 18% reported being current smokers.

Table 2 displays the bivariate analysis of all 12 independent variables or covariates (age, sex, race/ethnicity, employment status, marital status, education attained, annual household income, employment status binge drinking, smoking status, chronic disease, and geographic locale) by the study dependent variable—HSD. All of the covariates were categorical. For covariates with more than two factors, a chi square test for significance was calculated. For bifurcated covariates, odds ratios were used as the test statistic. Bivariate analysis revealed that each of the covariates had a statistically significant relationship with the dependent variable. Given these results, all of the independent variables were entered into the multivariate model tested.

The results of the first multivariate model tested are displayed in Table 3. The population for this model was all US veterans in 2014. All reference (comparison) categories are indicated in the table. Logistic regression analysis yielded that rural veterans had higher odds of having at least one HSD. This was also the case for Hispanic veterans, those reporting other/multirace/ethnicity, aged ≤64 years, earning <$75,000, less educated (<high school or at least high school), not married or coupled, current smokers, and binge drinkers. Also, adult veterans with no chronic disease had higher odds of having at least one HSD. Female sex and African American race were protective from at least one HSD.

Table 4 presents the results of the second multivariate model tested. All reference (comparison) categories are indicated in the table. The logistic regression analysis of rural US veterans in 2014 revealed that those who had higher odds of having at least one HSD were: 18–64 years of age, unemployed seeking employment, living in households with annual incomes lower than $75,000, without a university degree, not part of a married or unmarried couple, a current smoker, and/or a binge drinker within the last 30 days. Being female or African American was protective against having at least one HSD.

## 4. Discussion

This study sought to ascertain if the geographic locale of US veterans impacted their odds of experiencing at least one HSD. More specifically, we were interested in determining if rural residency of veterans was an independent risk factor for HSDs after controlling for a number of possibly contributing covariates. A number of notable findings emerged from this study, the first of which was that rural veterans had higher odds of having at least one HSD when compared to their urban counterparts. Furthermore, our results yielded that rural veterans ages 18 to 64 had greater odds of experiencing at least one HSD. This second finding was not dissimilar to that regarding all US veterans regardless of geographic locale. Our results also revealed that rural veterans with incomes less than $35,000 also had greater odds of experiencing at least one HSD, as did those without a university degree. Rural veterans who were current smokers and who were binge drinkers in the past 30 days also had higher odds of having at least one HSD.

These findings provide an epidemiological snapshot of rural US veterans experiencing at least one health service deficit in 2014. HSDs are not a small issue, given the difficulties of the VA in processing claims and providing healthcare for veterans that came to a head in 2014 [21]. Rural residency emerged as an independent risk factor for HSDs for veterans. Recent research has established that rural residency is an independent risk factor for adverse health-related issues for a number of specific diseases and conditions separate from veteran status [22]. The findings of this study provide further supporting evidence of the significance or importance of place in accounting for health, healthcare, and associated disparities.

That younger veterans had greater odds of having at least one HSD indicates that these are most likely veterans who have served in the Gulf-war era and the more recent Middle Eastern conflicts. All of the HSDs may be an issue for these veterans. For instance, there may be a common misperception that veterans are entitled to receive lifetime medical insurance and care. However, coverage for medical services is not guaranteed for all military veterans regardless of combat status [23]. While it is often cited that accessing services and availability of services may be a difficult issue for rural veterans, the reality may be more complicated than simple access. To receive veteran-related medical insurance and coverage, there is a prioritization system based on medical conditions related to military service [24]. Furthermore, there is an income-based adjustment for priority level for eligibility for services [23]. For veterans returning from a theater of combat, one must apply for healthcare coverage within five years after honorable or general discharge from active duty or risk losing eligible services [23,24].

Smoking and binge drinking are health-related risk behaviors. Their strong association with HSDs and rural veterans is worth noting and addressing in any interventions focused on reducing HSDs for this population. It may be the case that binge drinking is indicative of some measure of self-medication. Furthermore, engaging in these risky health behaviors may indicate that other similarly risky behaviors not measured in this study are also practiced.

Lower income and less educated rural veterans are a more vulnerable group of veterans. That these characteristics are also strongly associated with HSDs should be taken into consideration when developing any outreach or interventions aimed at reducing their HSDs.

It is also interesting and notable that African American race was protective both generally and in the instance of rural residency for HSDs. It may very well be the case that, in contrast to their other race and ethnicity counterparts, African American veterans have applied for, received, and used the healthcare benefits they are eligible for. These findings bear further examination.

Several potential limitations to this study should be noted. First, the survey is based on telephone-derived data and may lack representation because those who could not be reached by phone could not participate in the survey. For instance, persons of lower socioeconomic status may have been excluded because of poorer phone access. Widespread use of answering machines and caller identification now allow people to filter their phone calls, potentially leading to a passive refusal to participate in surveys such as the BRFSS. Nevertheless, call filtering is beyond the control of survey administrators and the vast majority of US residents live in households with telephones, which minimizes the bias of lack of phone access. Additionally, US cell phone numbers are now included in the pool of phones contacted for the survey ensuring the widest possible net being cast. A second limitation is that the survey used close-ended questions, which limit participants’ options to fully explain response choices. Nonetheless, the survey questions were worded such that the answer choices covered a wide range of response possibilities. A third, and related, limitation is that the answers are self-reported, which introduces the possibility of recall bias on the part of the survey participants. In addition to recall bias with self-reported data on health issues, there is the possibility of either under-reporting or over-reporting health concerns. Fourth, geographic locale is not provided for US veterans residing in Guam, Puerto Rico, or the US Virgin Islands. This may result in an under-reporting or under-identification of rural veterans. Finally, we were unable to identify a number of potentially important characteristics of the military veterans including combat status, location of services, deployment status, and length of active military service. Despite these limitations, there are a number of strengths worth noting. This is a population-based study analyzing data representative of the US population of veterans—rural and urban alike. Moreover, the data are surveillance data collected separately from any medical records or VA collected data.

## 5. Conclusions

The study described here fills identified epidemiological gaps in our knowledge regarding rural US military veterans and HSDs. Rural veterans have greater odds of having an HSD when compared to their urban counterparts. A major strength of the study is that non-medical and non-VA data were analyzed. The findings are not only interesting but important and should be used to inform interventions to reduce HSDs for rural veterans.

## Figures and Tables

**Table 1:** Description of US Veterans.

2014 BRFSS Data (Weighted *n* = 27,778,365)			
Variables and Factors		Frequency	Percent
**Geographic Locale**	Rural	2,905,663	20.4
	Non-Rural	11,323,343	79.6
**Respondents Sex**	Male	25,206,439	90.7
	Female	2,571,926	9.3
**Age**	18–64 Years	22,417,141	80.7
	65 Years and Older	5,361,224	19.3
**Race/Ethnicity**	Caucasian Non-Hispanic	20,491,361	75.4
	African American Non-Caucasian	3,229,867	11.9
	Hispanic	2,069,167	7.6
	Other/Multiracial Non-Hispanic	1,402,559	5.2
**Education Attained**	Less Than High School	1,776,697	6.4
	At Least High School	18,706,212	67.7
	At Least BA	7,131,746	25.8
**Marital Status**	Married or Part of Unmarried Couple	17,955,948	64.9
	Not Married or Part of Unmarried Couple	9,705,666	35.1
**Annual Household Income**	<$35,000	8,148,096	33.3
	$35,000 ≤ $75,000	8,698,137	35.5
	$75,000 And Higher	7,623,518	31.2
**Employment Status**	Employed	13,026,337	47.4
	Unemployed Seeking Employment	1,075,008	3.9
	Not Employed Not Seeking Employment	11,702,352	42.6
	Unable to Work	1,652,003	6.0
**Self-Reported Health Status**	Good to Excellent Health	22,457,186	81.1
	Fair to Poor Health	5,221,494	18.9
**Binge Drinking**	No Binge Drinking	22,009,387	85.3
	Binge Drinking	3,789,170	14.7
**Smoking Status**	Non-Smoker	21,897,016	82.2
	Current Smoker	4,736,563	17.8
**Health Insurance Status**	Have Health Insurance	26,064,558	94.2
	Do Not Have Health Insurance	1,612,250	5.8
**Personal Healthcare Provider**	Have Healthcare Provider	22,745,371	82.3
	Do Not Have Healthcare Provider	4,887,418	17.7
**Deferred Healthcare Because Of Cost**	Did Not Defer Care	25,730,431	92.8
	Deferred Care Because Of Cost	1,996,653	7.2
**Last Routine Medical Checkup**	Within Last 12 Months	22,067,917	79.9
	Longer Than 12 Months Ago	5,548,378	20.1
Health Service Deficits *	No Health Service Deficits	17,927,624	64.5
	At Least One Health Service Deficit	9,850,742	35.5
**BMI**	Not Obese	18,750,657	69.6
	Obese	8,175,188	30.4
**Arthritis**	No Arthritis	17,999,020	65.2
	Arthritis	9,603,728	34.8
**Depression in Lifetime**	No Depression	23,280,332	84.3
	Depression	4,351,672	15.7
**Diabetes**	No Diabetes	23,304,524	84.0
	Diabetes	4,435,093	16.0
Chronic Disease **	No Chronic Disease	10,443,692	39.2
	At Least One Chronic Disease	16,198,396	60.8

* Variable computed from the variable factors: do not have health insurance, do not have personal healthcare provider, last routine medical checkup longer than 12 months ago, in last 12 months deferred healthcare because of cost; ** Variable computed from the variable factors: obese, have arthritis, have lifetime depression, have diabetes.

**Table 2:** Bivariate Analysis with Dependent Variable Health Service Deficit *.

2014 BRFSS Data (weighted *n* = 27,778,365)				
Variables and Factors		Health Service Deficit (HSD)		Unadjusted Odds Ratio or P value for Chi Square Test
		% No Health Service Deficits	% At Least One Health Service Deficit	
Geographic Locale	Rural	19.6	22.6	OR = 1.140 (95 CI 1.137, 1.142)
	Non-Rural	80.4	77.4	Rural veterans had higher odds of having at least one HSD
Sex	Male	91.1	90.1	OR = 1.045 (95 CI 1.044, 1.046)
	Female	8.9	9.9	Male veterans had greater odds of not having at least one HSD
Age Range	18–64 Years	70.8	91.5	OR = 2.558 (95 CI 2.557, 2.559)
	65 Years and older	29.2	8.5	Veterans aged 18–64 years had greater odds of having at least one HSD
Race/Ethnicity	Caucasian Non-Hispanic	77.5	71.4	*p* = 0.000 for chi square test indicating that there is a strong association between race/ethnicity and HSDs
	African American Non-Hispanic	11.9	11.9	
	Hispanic	6.5	9.7	
	Other Non-Hispanic	4.2	6.9	
Education Attained	Less than High School	5.8	7.7	*p* = 0.000 for chi square test indicating that there is a strong association between education and HSDs
	At Least High School	65.6	71.7	
	At Least BA	28.7	20.7	
Marital Status	Married Or Part Of Unmarried Couple	69.7	56.2	OR = 1.244 (95 CI 1.243, 1.245)
	Not Married Or Part Of Unmarried Couple	30.3	43.8	Veterans who were married or part of an unmarried couple had greater odds of not having at least one HSD
Annual Household Income	<$35,000	29.4	40.5	*p* = 0.000 for chi square test indicating that there is a strong association between annual household income and HSDs
	$35,000–<$75,000	36.9	33.0	
	$75,000 And Higher	33.7	26.5	
Employment Status	Employed	41.2	58.8	*p* = 0.000 for chi square test indicating that there is a strong association between employment status and HSDs
	Unemployed Seeking Employment	2.5	6.5	
	Not Employed Not Seeking Employment	50.9	27.5	
	Unable To Work	5.4	7.1	
Self-Reported Health Status	Good to Excellent Health	80.6	82.0	OR = 1.061 (95 CI 1.060, 1.063)
	Fair to Poor Health	19.4	18.0	Veterans self-reporting good to excellent health had higher odds of having at least one HSD
Binge Drinking	No Binge Drinking	88.1	80.1	OR = 1.280 (95 CI 1.278, 1.281)
	Binge Drinking	11.9	19.9	Veterans with no binge drinking had higher odds of having no HSDs
Smoking Status	Non-Smoker	86.4	74.6	OR = 1.370 (95 CI 1.369, 1.371)
	Current Smoker	13.6	25.4	Non-smoking veterans had higher odds of having no HSDs
Chronic Disease **	No Chronic Disease	34.4	48.1	OR = 1.436 (95 CI 1.435, 1.438)
	At Least One Chronic Disease	65.6	51.9	Veterans with no chronic disease had higher odds of having at least one HSD

* Variable computed from the variable factors: do not have health insurance, do not have personal healthcare provider, last routine medical checkup longer than 12 months ago, in last 12 months deferred healthcare because of cost; ** Variable computed from the variable factors: obese, have arthritis, have lifetime depression, have diabetes.

**Table 3:** Logistic Regression Analysis of US Veterans with Dependent Variable = Health Service Deficits *.

2014 BRFSS Data				
Variables and Factors		Adjusted Odds Ratio (AOR)		
		AOR	Lower CI	Upper CI
Geographic Locale	Rural	1.169	1.167	1.170
	Non-Rural	-- *		
Race/Ethnicity	Caucasian Non-Hispanic	-- *		
	African American Non-Hispanic	0.755	0.754	0.756
	Hispanic	1.392	1.389	1.394
	Other Non-Hispanic	1.326	1.323	1.328
Respondents Sex	Male	-- *		
	Female	0.815	0.814	0.816
Age	18–64 Years	3.290	3.285	3.294
	65 Years And Older	-- *		
Annual Household Income	<$35,000	2.346	2.342	2.349
	$35,000 ≤ $75,000	1.408	1.406	1.409
	$75,000 And Higher	-- *		
Employment Status	Employed	1.550	1.547	1.553
	Unemployed Seeking Employment	2.426	2.420	2.433
	Not Employed Not Seeking Employment	1.203	1.200	1.205
	Unable To Work	-- *		
Education Attained	Less Than High School	1.464	1.461	1.467
	At Least High School	1.089	1.088	1.091
	At Least BA	-- *		
Marital Status	Married Or Part Of Unmarried Couple	-- *		
	Not Married Or Part Of Unmarried Couple	1.124	1.123	1.125
Smoking Status	Non-Smoker	-- *		
	Current Smoker	1.360	1.358	1.361
Binge Drinking	No Binge Drinking	-- *		
	Binge Drinking	1.271	1.269	1.273
Self-Reported Health Status	Good To Excellent Health	-- *		
	Fair To Poor Health	1.052	1.050	1.053
Chronic Disease **	No Chronic Disease	1.382	1.380	1.383
	At Least One Chronic Disease	-- *		

* Variable computed from the variable factors: do not have health insurance, do not have personal healthcare provider, last routine medical checkup longer than 12 months ago, in last 12 months deferred healthcare because of cost; ** Variable computed from the variable factors: obese, have arthritis, have lifetime depression, have diabetes.

**Table 4:** Logistic Regression Analysis of Rural US Veterans with Dependent Variable = Health Service Deficits *.

2014 BRFSS Data				
Variables and Factors		Adjusted Odds Ratio and 95% CI		
		OR	Lower CI	Upper CI
Race/Ethnicity	Caucasian Non-Hispanic	-- *		
	African American Non-Hispanic	0.777	0.766	0.788
	Hispanic	1.076	1.056	1.097
	Other/Multiracial Non- Hispanic	1.448	1.429	1.469
Employment Status	Employed	1.002	.990	1.014
	Unemployed Seeking Employment	1.823	1.788	1.858
	Not Employed Not Seeking Employment	0.739	0.731	0.749
	Unable To Work	-- *		
Annual Household Income	<$35,000	1.659	1.644	1.675
	$35,000 ≤ $75,000	1.164	1.154	1.173
	$75,000 And Higher	-- *		
Education Attained	Less Than High School	1.700	1.679	1.721
	At Least High School	1.112	1.103	1.121
	At Least BA	-- *		
Age	18–64 Years	1.995	1.979	2.010
	65 Years And Older	-- *		
Marital Status	Married Or Part Of Unmarried Couple	-- *		
	Not Married Or Part Of Unmarried Couple	1.263	1.254	1.271
Chronic Disease **	No Chronic Disease	1.340	1.331	1.348
	At Least One Chronic Disease	-- *		
Smoking Status	Non-Smoker	-- *		
	Current Smoker	1.486	1.475	1.498
Binge Drinking	No Binge Drinking	-- *		
	Binge Drinking	1.410	1.397	1.422
Self-Reported Health Status	Good To Excellent Health	-- *		
	Fair To Poor Health	1.000	0.992	1.007
Respondents Sex	Male	-- *		
	Female	0.887	0.877	0.898

* Variable computed from the variable factors: do not have health insurance, do not have personal healthcare provider, last routine medical checkup longer than 12 months ago, in last 12 months deferred healthcare because of cost; ** Variable computed from the variable factors: obese, have arthritis, have lifetime depression, have diabetes.
